# A retrospective study of prognostic factors and prostate-specific antigen dynamics in Japanese patients with metastatic hormone-sensitive prostate cancer who received combined androgen blockade therapy with bicalutamide

**DOI:** 10.1007/s10147-024-02597-x

**Published:** 2024-08-17

**Authors:** Yu Tashiro, Shusuke Akamatsu, Kentaro Ueno, Toshiyuki Kamoto, Naoki Terada, Takuya Hida, Ryoma Kurahashi, Tomomi Kamba, Atsushi Saito, Takumi Lee, Satoshi Morita, Takashi Kobayashi

**Affiliations:** 1 Department of Urology, Japanese Red Cross Otsu Hospital, 1 Chome-1-35 Nagara, Otsu, Shiga 520-0046 Japan; 2 Department of Urology, Kyoto University Graduate School of Medicine, 54 Shogoinkawahara-cho, Sakyo-ku, Kyoto, 606-8507 Japan; 3 Department of Urology, Nagoya University Graduate School of Medicine, 2 Chome-1-10 Kitachikusa, Chikusa Ward, Nagoya, Aichi 464-0083 Japan; 4 Department of Biomedical Statistics and Bioinformatics, Kyoto University Graduate School of Medicine, Yoshidakonoecho, Sakyo Ward, Kyoto, 606-8303 Japan; 5 Department of Urology, University of Miyazaki, Kihara-5200 Kiyotakecho, Miyazaki, 889-1601 Japan; 6 Department of Urology, University of Fukui, 3 Chome-9-1 Bunkyo, Fukui, 910-0017 Japan; 7 Department of Urology, Graduate School of Medical Sciences, Kumamoto University, 2 Chome-40-1 Kurokami, Chuo Ward, Kumamoto, 860-0862 Japan; 8 Astellas Pharma Inc, 2-5-1, Nihonbashi-Honcho, Chuo-Ku, Tokyo, 103-8411 Japan

**Keywords:** Bicalutamide, Combined androgen blockage, Japan, Metastatic hormone-sensitive prostate cancer

## Abstract

**Background:**

This retrospective observational study explored the therapeutic potential of combined androgen blockade (CAB) with bicalutamide (Bic-CAB) as an initial treatment for metastatic hormone-sensitive prostate cancer (mHSPC) in Japan.

**Methods:**

The electronic health records of 159 patients with mHSPC from three Japanese institutions who received initial treatment with Bic-CAB between 2007 and 2017 were analyzed. The time to prostate-specific antigen (PSA) progression, duration of Bic-CAB treatment, and overall survival (OS), with various definitions for PSA progression, were assessed. A multivariate Cox proportional hazards model was constructed using clinical parameters to predict time to the end of Bic-CAB treatment and OS.

**Results:**

The median observation period was 46.4 months, and the median age of patients at diagnosis was 71 years. A total of 46.5% patients experienced PSA progression with a median survival duration of 29 months (according to Prostate Cancer Clinical Trials Working Group 3 criteria), and 49.1% patients achieved a PSA nadir < 0.2 ng/mL in a median time of 4.7 months. When stratified by PSA nadir and PSA change, patients at low risk for disease progression with a small PSA change due to low initial PSA had a 5-year OS of 100% and a 10-year OS of 75%. The OS during the observation period was 72.9 months.

**Conclusion:**

These findings highlight the potential effect of Bic-CAB in patients with mHSPC who were at low risk for disease progression. Initial treatment with Bic-CAB and adjusting treatment early based on PSA dynamics may be a reasonable treatment plan for these patients.

## Introduction

Prostate cancer is the second most common cancer and fifth leading cause of cancer-related mortality in men globally with over 1.4 million new cases in 2020 [1]. Among Japanese men, the incidence of prostate cancer has shown a rapid increase, with an incidence rate of 154.3 cases per 100,000 population and a mortality rate of 21.3 patients per 100,000 population [2, 3]. Androgen-deprivation therapy (ADT) has been the conventional treatment for patients with metastatic prostate cancer [4, 5]. However, in patients with metastatic hormone-sensitive prostate cancer (mHSPC) who are initially sensitive to ADT, the disease is likely to transition to metastatic castration-resistant prostate cancer (mCRPC), which progresses clinically, radiographically, or biochemically despite castrate levels of serum testosterone [6]. Therefore, delaying the onset of mCRPC remains the primary treatment goal for patients with mHSPC [7].

In recent years, novel androgen receptor signaling inhibitors (ARSIs) have gained widespread recognition for their role in improving overall survival (OS) in patients with mHSPC [8, 9]. Treatment guidelines recommend a combination of ARSI and ADT as initial treatment for mHSPC [10]. The therapeutic efficacy of ARSIs has been well established in Japanese patients with prostate cancer [11]. Nonetheless, due to the heterogeneity of mHSPC, ARSIs alone may not consistently provide substantial improvements in patient outcomes, and treatment intensification with a combination of ARSI and docetaxel as initial treatment has demonstrated potential benefits [12, 13]. Another approach involves de-escalation of systemic treatment by using radiotherapy on primary and/or metastatic lesions, particularly in cases with a limited number of metastases [14]. In light of this evolving treatment landscape, although ARSIs are the standard of care for mHSPC, risk stratification in patients with mHSPC is crucial to provide an optimal initial therapeutic regimen [10].

Given the prolonged natural history of prostate cancer, the duration of response among Japanese patients treated with ARSI + ADT for mHSPC is long [15]. Combined androgen blockade (CAB) therapy with bicalutamide (Bic-CAB) is a potential initial treatment approach for mHSPC in Japan, due to (i) the high prevalence of mHSPC in the older population (> 80 years old), in whom treatment with ARSIs has been associated with frequent adverse events, and (ii) the economic advantages associated with this treatment regimen [16].

While Bic-CAB as initial treatment may be less efficacious than ARSIs, it may result in a favorable long-term prognosis when administered to the right patients. Studies conducted in Japan have underlined certain clinical parameters as predictors of OS in patients with mHSPC [17]; these include extent of disease (EOD) score ≥ 2, presence of liver metastasis, lactate dehydrogenase (LDH) > 250 U/L, and a primary Gleason score of 5 [17]. Additionally, prostate-specific antigen (PSA) dynamics after treatment initiation have been well recognized as a predictive surrogate for OS [18], whereby a lower nadir PSA level is associated with delayed progression to mCRPC and longer OS in patients who concomitantly receive ARSIs after the initiation of primary ADT [19, 20].

In this observational study, we used a combination of baseline clinical characteristics and PSA dynamics after treatment initiation to identify patients with mHSPC who were expected to achieve long-term survival after receiving treatment with Bic-CAB.

## Materials and methods

### Study design and data source

This was a retrospective analysis of the electronic health records of 159 patients with metastatic prostate cancer who received initial treatment with Bic-CAB at three institutions in Japan (Kyoto University Hospital, Kumamoto University Hospital, and University of Miyazaki Hospital) from January 2007 to December 2017. Bic-CAB was defined as treatment with bicalutamide (80 mg /day) in combination with a luteinizing hormone–releasing hormone (LH-RH) (gonadotropin-releasing hormone [GnRH]) agonist, LH-RH antagonist, or surgical castration.

All patients with mHSPC who received Bic-CAB as initial treatment were included in the study. The index date was defined as the date bicalutamide was first prescribed to the target patient in the participating facility during the study period, and the survey period for data extraction ended on 30 September 2019. The participating study sites extracted information on the efficacy of Bic-CAB from the medical records of patients from the index date until the final visit. Patient demographics and prostate cancer treatment history were also collected. PSA data were captured at baseline and at 3 and 6 months after the index date, including PSA nadir data during Bic-CAB treatment.

### Study population

The inclusion criteria were as follows: confirmed diagnosis of metastatic prostate cancer involving at least one non-regional lymph node metastasis as confirmed by computed tomography, magnetic resonance imaging, or bone scintigraphy before the index date; EOD for objective assessment [21]; Bic-CAB as the first-line treatment; and PSA measured at baseline and at least two instances at the time of diagnosis and during Bic-CAB. Patients who had received ADT monotherapy for longer than 3 months prior to the index date, had a total follow-up period of less than 6 months, or were treated with local therapy for prostate cancer (surgery or radiation) during the Bic-CAB treatment period were excluded from the study.

### Study outcomes

The primary study outcomes were time to PSA progression, duration of Bic-CAB treatment, and OS. PSA progression was defined using the following clinical definitions:

1. A continuous increase in PSA at measurement intervals of at least 1 week with a minimum starting value of 1.0 ng/mL based on the recommendations of the Prostate Cancer Working Group 3 (PCWG3) [22].
2. A continuous increase in PSA at measurement intervals of at least 1 week with a minimum starting value of 2.0 ng/mL (a pre-treatment progression criterion of PCWG2) [22, 23].
3. An increase in PSA value by ≥ 25% with an absolute increase of ≥ 2.0 ng/mL from nadir (a progression criterion of PCWG2) [22, 23].
4. An increase in PSA ≥ 25% from nadir.
5. Three continuous increases in PSA at measurement intervals of at least 1 week.

The end of Bic-CAB treatment was defined as discontinuation of bicalutamide regardless of the PSA level. Nadir was defined as the lowest PSA value obtained at or after baseline. The cutoff value for PSA nadir was 0.2 ng/mL.

### Statistical analysis

Statistical analysis was performed using SAS Version 9.4. Medians and 95% confidence intervals (CIs) were calculated using the Kaplan–Meier method for time to PSA progression, duration of Bic-CAB treatment, and OS. A *p* value less than 0.05 was considered statistically significant. A multivariate Cox proportional hazards model was constructed using clinical parameters such as Eastern Cooperative Oncology Group (ECOG) performance status (PS), log(initial PSA), hemoglobin, albumin, alkaline phosphatase, lactate dehydrogenase, Gleason score sum score, liver metastasis, and EOD score to investigate the prediction models for time to the end of Bic-CAB treatment.

Bic-CAB failure was defined as the criterion for discontinuation of Bic-CAB. The PSA levels in patients receiving Bic-CAB on and after the index date were summarized. While the PSA detection sensitivities differed among the participating sites, if the PSA reached undetectable levels, the PSA nadir was considered to be 0.2 ng/mL in consideration of clinical significance.

### Ethics statement

This observational study was approved by the institutional review board of each institution.

## Results

### Patient characteristics

A total of 159 patients were enrolled in the study. The median (inter quartile range, [IQR]) observation period was 46.4 months (27.3–78.6), and the median age (IQR) at diagnosis was 71 years (49–88). Most patients (89.3%, n = 142) had an ECOG PS of 0–1. At diagnosis, 52 patients (32.7%) experienced pain, and the median (IQR) initial PSA (iPSA) and LDH levels were 224.5 ng/mL (59.8–662.0) and 188 U/L (163.0–229.0), respectively. A total of 67 patients (42.1%) were in Gleason grade (GG) group 4, and 74 patients (46.5%) were in GG group 5. Bone metastases were observed in 150 patients (94.3%); metastases of the distant lymph nodes, in 52 patients (32.7%); and visceral metastases, in 23 patients (14.5%). The EOD score was 0–1 in 80 patients (50.4%) and 2–4 in 76 patients (47.7%). The tumor volume according to the CHAARTED criteria was defined as “high volume of metastasis” in 91 patients (57.2%) and “low volume of metastasis” in 68 patients (42.8%). By stratifying patients by independent prognostic factors (EOD score ≥ 2, presence of liver metastasis, LDH > 250 U/L, and a primary Gleason score of 5) according to the Kyoto University risk grading system [17], 61 patients (38.4%) fell into the low-risk group, 48 patients (30.2%) into the intermediate-risk group, and 40 patients (25.2%) into the high-risk group (Table 1).

**Table 1:** Baseline characteristics of study participants

Baseline characteristics	N = 159
Age in years, median (IQR)	71 (49–88)
ECOG PS, *n* (%)	
0–1	142 (89.3)
2	12 (7.5)
3–4	4 (2.5)
N/A	1 (0.6)
Pain	
Present	52 (32.7%)
Absent	107 (67.3%)
Laboratory data, median (IQR)	
Initial PSA, ng/mL	224.5 (59.8–662)
Hemoglobin, g/dL	13.5 (12.2–14.7)
Albumin, g/dL	4.0 (3.7–4.2)
Alkaline phosphatase, U/L	361 (227–839)
Lactate dehydrogenase, U/L	188 (163–229)
Gleason grade group, *n* (%)	
1–3	17 (10.7%)
4	67 (42.1%)
5	74 (46.5%)
N/A	1 (0.6%)
EOD score, *n* (%)	
0–1	80 (50.4%)
2–4	76 (47.7%)
Metastatic lesion, *n *(%)^a^	
Distant lymph node	52 (32.7%)
Bone	150 (94.3%)
Visceral	23 (14.5)
Prognostic risk according to Kyoto University risk grading system, *n* (%)	
Low risk	61 (38.4%)
Intermediate risk	48 (30.2%)
High risk	40 (25.2%)
N/A	10 (6.2%)
Tumor volume based on CHAARTED criteria	
High-volume metastasis	91 (57.2%)
Low-volume metastasis	68 (42.8%)

*CHAARTED* Chemohormonal Therapy in Metastatic Hormone-Sensitive Prostate Cancer: Long-Term Survival Analysis of the Randomized Phase III E3805 CHAARTED Trial, *ECOG PS* Eastern Cooperative Oncology Group performance status, *EOD* extent of disease, *IQR* interquartile range, *N/A* not applicable, *PSA*, prostate-specific antigen

^a^Multiple metastases in a single patient were counted separately

### Time to Bic-CAB failure

During the observation period, Bic-CAB failure was observed in 117 of the 159 patients, and the median (IQR) time to Bic-CAB failure was 19.9 (14.0–28.4) months. Multivariate Cox regression analysis revealed that the time to Bic-CAB failure was associated with ECOG PS ≥ 2, LDH level > 250 U/L, an International Society of Urological Pathology grading group (ISUP GG) (sum score) 5, and an EOD score ≥ 2 (Table 2).

**Table 2:** Univariate and multivariate Cox regression analysis of time to Bic-CAB failure

		Univariate			Multivariate	
Baseline characteristics	N = 159	Hazard ratio (95% CI)	*p* value	N = 147	Hazard ratio (95% CI)	*p* value
Age in years						
≥ 65	125	1				
< 65	34	1.09 (0.71–1.67)	0.687			
ECOG PS						
0–1	142	1		132	1	
≥ 2	16	1.68 (0.96–2.95)	0.068	15	1.93 (1.04–3.6)	0.038
Log(initial PSA)	159	1.11 (1.01–1.21)	0.026	147	1.02 (0.92–1.14)	0.670
Hemoglobin (g/dL)						
> 12.0	119	1		115	1	
≤ 12.0	35	1.66 (1.07–2.56)	0.023	32	0.62 (0.33–1.17)	0.141
Albumin (g/dL)						
> 3.9	89	1		86	1	
≤ 3.9	64	1.50 (1.04–2.18)	0.032	61	1.23 (0.82–1.85)	0.320
Alkaline phosphatase (U/L)						
≤ 500	96	1		92	1	
> 500	57	2.19 (1.51–3.18)	< 0.001	55	1.28 (0.78–2.11)	0.329
Lactate dehydrogenase (U/L)						
≤ 250	122	1		118	1	
> 250	31	3.38 (2.14–5.32)	< 0.001	29	2.36 (1.12–4.58)	0.011
ISUP GG						
1–4	84	1		79	1	
5	74	1.88 (1.30–2.72)	< 0.001	68	1.67 (1.12–2.5)	0.012
Liver metastasis						
No	157	1		145	1	
Yes	2	7.82 (1.83–33.34)	0.005	2	3.63 (0.80–16.50)	0.095
EOD score						
0–1	80	1		74	1	
2–4	76	2.77 (1.89–4.04)	< 0.001	73	2.15 (1.33–3.47)	0.002

*Bic-CAB* combined androgen blockade with bicalutamide, *CI* confidence interval, *ECOG PS* Eastern Cooperative Oncology Group performance status, *EOD* extent of disease, *ISUP GG* International Society of Urological Pathology grade group, *PSA* prostate-specific antigen

### Time to PSA progression

A total of 74 patients (46.5%) experienced an event of PSA progression as per definition 1, in a median (95% CI) time of 29.0 months (17.1–57.9). The number of patients who experienced an event of PSA progression as per definition 2 was 62 (39%), in a median (IQR) time of 60.6 months (26.1–90.4); as per definition 3, 69 patients (43.4%) experienced a PSA progression event in a median (95% CI) time of 35.7 months (22.5–89.2). As per definition 4, 103 patients (64.8%) experienced a PSA progression event, in a median (95% CI) time of 17.8 months (12.9–23.6); as per definition 5, 67 patients (42.1%) experienced a PSA progression event, at a median (IQR) time of 34.0 months (25.1–50.4) (Table 3).

**Table 3:** Time to PSA progression

Definition of PSA progression	Event	Censored	Time to PSA progression (months)	
	(N = 159)		Median	95% CI
Definition 1	74	85	29.0	17.1–57.9
Definition 2	62	97	60.6	26.1–90.4
Definition 3	69	90	35.7	22.5–89.2
Definition 4	103	56	17.8	12.9–23.6
Definition 5	67	92	34.0	25.1–50.4

*CI* confidence interval; PSA, prostate-specific antigen

Definition 1: PSA is continuously elevated at measurement intervals of 1 week or more (measured value is ≥ 1.0 ng/mL at progression)

Definition 2: PSA is continuously elevated at measurement intervals of 1 week or more (measured value is ≥ 2.0 ng/mL at progression)

Definition 3: PSA is elevated ≥ 25% from the nadir, and the range of increase is ≥ 2.0 ng/mL

Definition 4: PSA is elevated ≥ 25% from nadir

Definition 5: PSA is elevated at three consecutive measurements at measurement intervals of 1 week or more

### Overall survival

A total of 78 of the 159 (49.1%) patients died during the observation period. Median OS (IQR) was 72.9 months (58.2–114.7). Multivariate Cox regression analysis revealed that OS was associated with EOD score 2–4 (Table 4).

**Table 4:** Univariate and multivariate Cox regression analysis of OS

		Univariate		Multivariate		
Baseline characteristics	N = 159	Hazard ratio (95% CI)	*p* value	N = 147	Hazard ratio (95% CI)	*p* value
Age in years						
≥ 65	125	1				
< 65	34	1.04 (0.63–1.74)	0.868			
ECOG PS						
0–1	142	1		132	1	
≥ 2	16	1.29 (0.64–2.59)	0.469	15	1.19 (0.53–2.66)	0.679
Log(initial PSA)	159	1.06 (0.94–1.19)	0.341	147	0.93 (0.81–1.08)	0.368
Hemoglobin (g/dL)						
> 12.0	119	1		115	1	
≤ 12.0	35	2.39 (1.47–3.89)	< 0.001	32	1.35 (0.67–2.73)	0.395
Albumin (g/dL)						
> 3.9	89	1		86	1	
≤ 3.9	64	1.72 (1.10–2.69)	0.017	61	1.56 (0.95–2.56)	0.082
Alkaline phosphatase (U/L)						
≤ 500	96	1		92	1	
> 500	57	2.35 (1.50–3.67)	< 0.001	55	1.22 (0.63–2.34)	0.555
Lactate dehydrogenase (U/L)						
≤ 250	122	1		118	1	
> 250	31	3.72 (2.27–6.11)	< 0.001	29	1.79 (0.85–3.75)	0.124
ISUP GG						
1–4	84	1		79	1	
5	74	1.96 (1.24–3.09)	0.004	68	1.36 (0.81–2.29)	0.242
Liver metastasis						
No	157	1		145	1	
Yes	2	3.76 (0.91–15.44)	0.005	2	1.73 (0.37–8.03)	0.484
EOD score						
0–1	80	1		74	1	
≥ 2	76	2.88 (1.79–4.63)	< 0.001	73	2.13 (1.13–4.03)	0.020

*CI* confidence interval, *ECOG PS* Eastern Cooperative Oncology Group performance status, *EOD* extent of disease, *ISUP GG* International Society of Urological Pathology grade group, *PSA* prostate-specific antigen, *OS* overall survival

### OS stratified by PSA nadir and PSA change

**Fig. 1:**
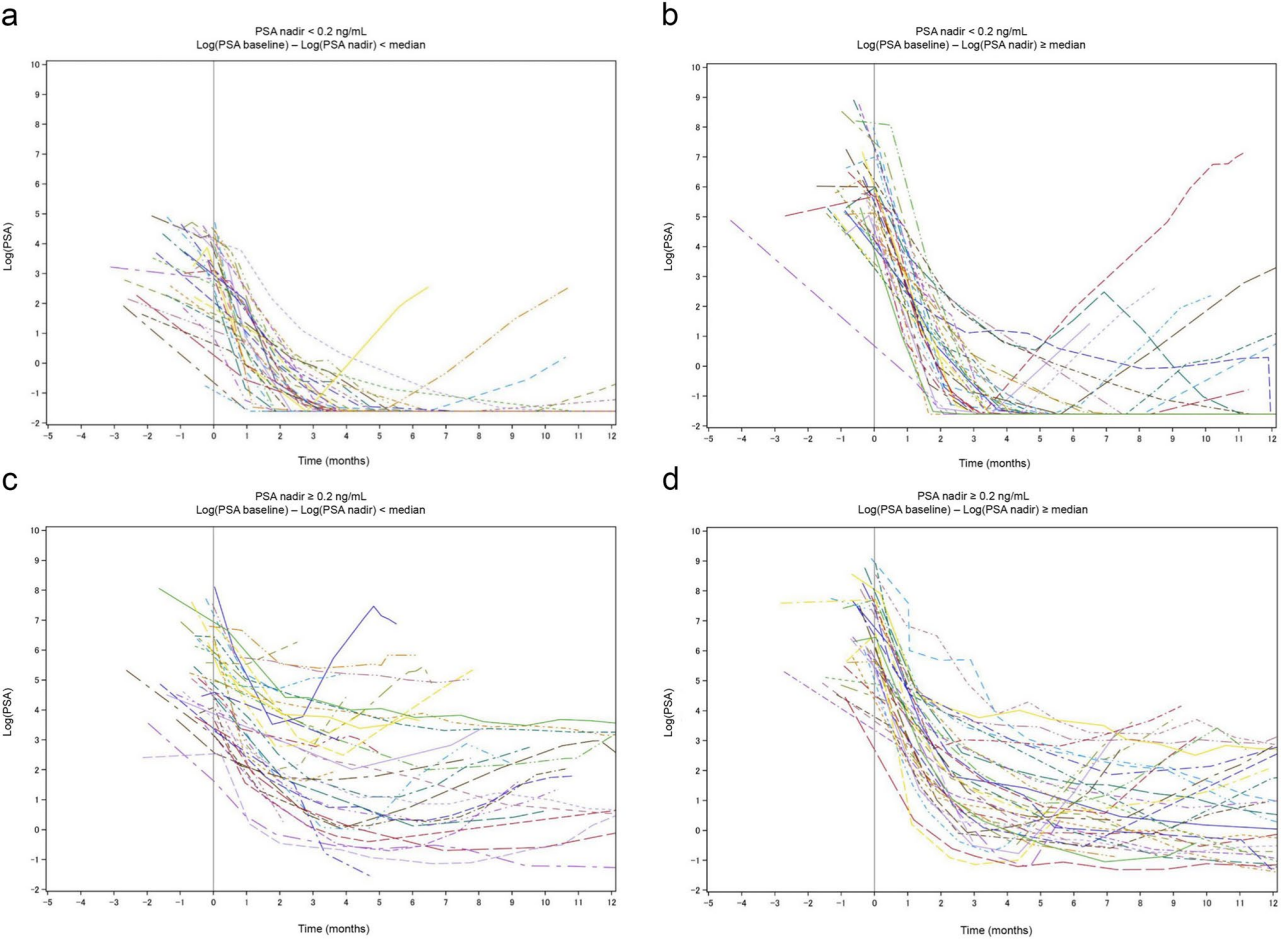

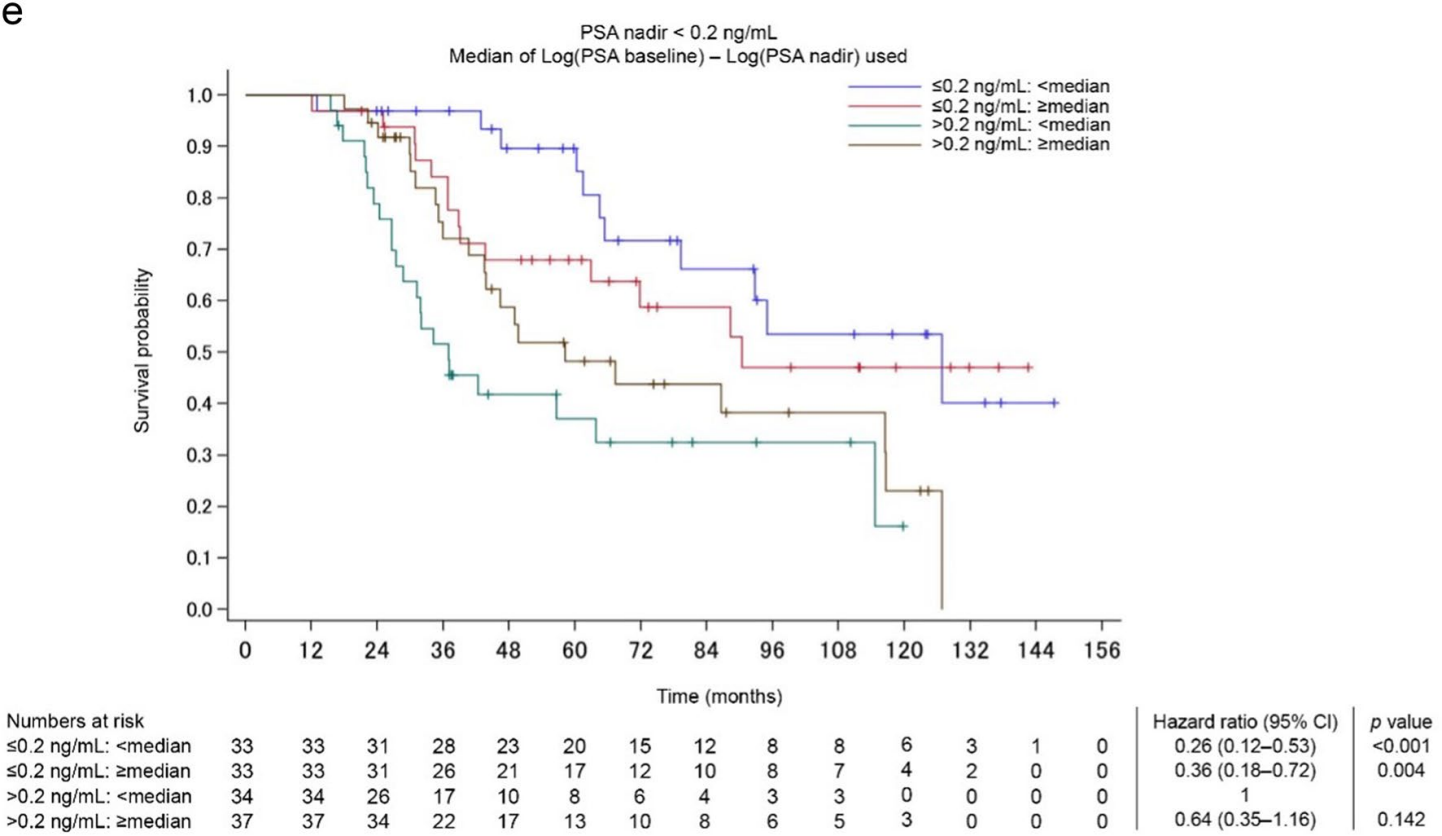
**a–e**: **a** PSA dynamics in patients with PSA nadir < 0.2 ng/mL and small PSA changes (log[initial PSA] − log[PSA nadir] < median). Except in a very few cases, PSA remains low. **b** PSA dynamics in patients with PSA nadir < 0.2 ng/mL and substantial PSA changes (log[initial PSA] − log[PSA nadir] ≥ median). In half of these cases, a PSA rebound is observed after the PSA nadir has been reached. **c** PSA dynamics in patients with PSA nadir ≥ 0.2 ng/mL and small PSA changes (log[initial PSA] − log[PSA nadir] < median). A temporary PSA response is observed, but in many cases, there is no sustained PSA decrease. **d** PSA dynamics in patients with PSA nadir ≥ 0.2 ng/mL and substantial PSA changes (log[initial PSA] − log[PSA nadir] ≥ median). As in **c**, there is a temporary PSA response but no sustained PSA decrease. **e** OS stratified based on the achievement of PSA nadir < 0.2 ng/mL and the range of change from before to after treatment

**Fig. 2:**
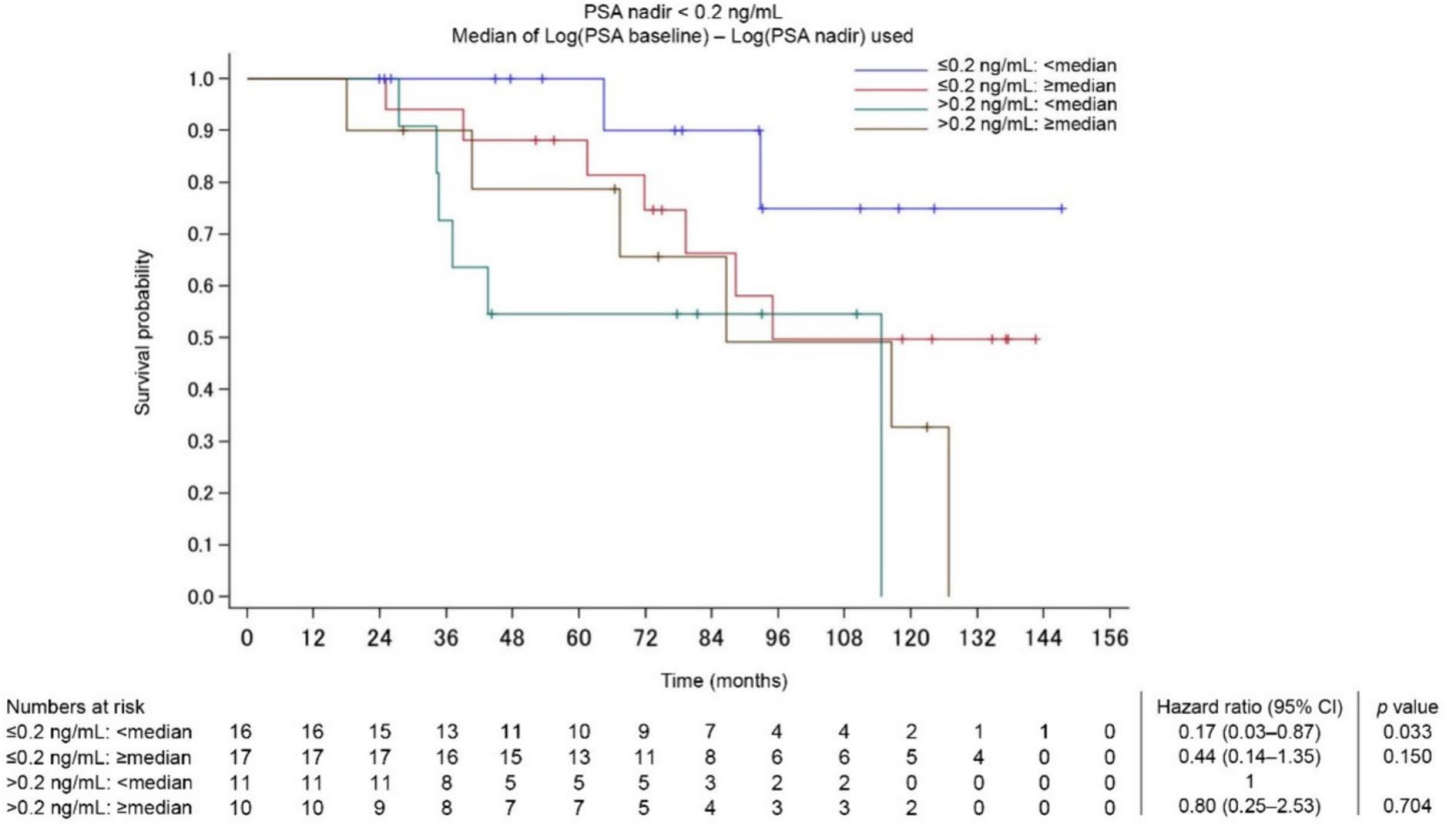
OS stratified by PSA nadir and PSA change in low-risk group as per Kyoto University risk grading system

A total of 78 patients (49.1%) achieved PSA nadir < 0.2 ng/mL in a median (IQR) time of 4.7 months (4.1–5.1). OS was divided into four groups based on PSA nadir and PSA change [log(initial PSA) − log(PSA nadir)]. Figure 1a and b show the PSA dynamics for patients who achieved PSA nadir < 0.2 ng/mL, while Fig. 1c and d show the PSA dynamics for those who did not. Among patients who achieved PSA nadir < 0.2 ng/mL, many with small PSA changes, reflecting low initial PSA, showed a sustained PSA response. On the other hand, even when PSA nadir < 0.2 ng/mL was achieved, half of the patients with substantial PSA changes, reflecting high initial PSA, showed PSA rebound within 12 months of treatment. OS stratified by PSA nadir < 0.2 ng/mL and the range of PSA change from before to after treatment are shown in Fig. 1e. An additional analysis of changes in OS over time was conducted based on achievement of PSA nadir and the range of PSA change in the low-risk group identified using the Kyoto University risk grading system, where Bic-CAB may be considered a potential option even though the current standard therapy is ARSI. Figure 2 shows the Kaplan–Meier curves of OS (over time in months) for the different PSA nadir and PSA change groups in the Kyoto classification of low-risk cases. Patients who achieved PSA nadir < 0.2 ng/mL with a small PSA change due to low iPSA had a 5-year OS probability of 100% and a 10-year OS probability of 75% (Fig. 2).

## Discussion

This observational study included 159 Japanese patients with mHSPC who received Bic-CAB as the initial treatment. The PSA progression and OS were assessed, with a focus on PSA dynamics. The PSA nadir values after treatment initiation were also assessed, which enabled identification of the clinical characteristics of patients who could achieve a good long-term prognosis with Bic-CAB. Time to PSA progression was evaluated using five different published definitions, and it was noted that an increase in PSA ≥ 25% from nadir was the closest to time to Bic-CAB failure which may suggest the optimal time point to consider change of treatment.

The characteristics of patients in this study were similar to those in previous studies: in patients with mHSPC who received CAB and those with newly diagnosed metastatic prostate cancer initially treated with ADT, the median age was 73 years; the Gleason score was approximately 8–10 in 86% patients, and bone metastases were the most common metastatic lesions [24, 25]. This indicates that the current study cohort is highly representative of Japanese patients with mHSPC.

Previous studies have described the prognostic and risk factors associated with mHSPC treatment in Japan [24]. Miyazawa et al. identified ECOG performance status, hemoglobin levels, LDH levels, EOD, visceral metastasis, and PSA response after 3 months of treatment as significant predictors of OS in patients with mHSPC treated with CAB [24]. The current study found a good prognosis in patients in the low-risk group of the Kyoto University risk grading system. However, some of these patients did not show a favorable treatment response, suggesting a limited capability to predict mHSPC prognosis based on baseline characteristics at diagnosis alone.

In the present study, we sought to explore PSA dynamics associated with survival outcomes. This included an exploratory analysis, in which we examined several parameters focusing on the extent and speed of PSA change and identified “log(iPSA) − log(PSA nadir)” as the best parameter to explain OS. In addition to the risk model reported previously [15], the status of achievement of PSA nadir < 0.2 ng/mL and the range of PSA change before and after treatment were assessed to identify patients with a prognosis of a 5-year survival of 100% and a 10-year survival of 75%. These findings suggest that patients who achieved PSA nadir < 0.2 ng/mL with a small PSA change, which reflects a low iPSA level, may be expected to have a good long-term prognosis with Bic-CAB. They are in line with a study by Sasaki et al. [26] that reported a PSA nadir < 0.2 ng/mL and a prolonged time to PSA nadir following primary ADT to be early predictors of longer survival in patients with prostate cancer and bone metastasis [26]. Although the clinical significance of iPSA in mHSPC has not yet been fully characterized, a high iPSA level is reportedly associated with a lower duration of response to initial ADT [27]. According to the current study, high iPSA levels may have a negative effect on the duration of response to Bic-CAB as an initial treatment. Furthermore, the PSA nadir achievement rate of 49% observed in the current study is higher than the previously reported PSA nadir achievement rate of 14.5% reported with ADT alone [20].

The present findings suggest that Bic-CAB could be considered as an initial treatment approach for patients who are in the low-risk group of the Kyoto University risk grading system and have a low PSA level at diagnosis. These patients could be initiated on Bic-CAB and switched to an ARSI if the PSA nadir of < 0.2 ng/mL is not achieved. Additionally, it is important to note that this study included patients who discontinued bicalutamide when PSA increased by 25% from nadir, even before meeting the PCWG2 or PCWG3 criteria for PSA progression, which are generally followed in clinical studies.

The findings suggest the possibility of using Bic-CAB as the initial systemic treatment in certain low-risk patients. Given the evolving landscape of mHSPC treatment and considering that radiation of primary lesions is a current standard treatment regimen in patients with a small number of metastases, the combination of Bic-CAB with radiation of primary lesions may offer a potential therapeutic avenue for certain patients with mHSPC in Japan [22, 23].

The findings of this study should be interpreted in the context of certain limitations. First, the retrospective observational design introduced a potential for selection biases, as standardized criteria for treatment changes were not uniformly applied. The multivariate analysis included a substantial number of factors but a limited number of events. Therefore, critical reassessment of the multivariate analysis method is important, considering the independence of each individual factor. Lastly, the study was conducted in a specific patient population in Japan, and this should be accounted for when considering the applicability of the findings to broader patient cohorts and healthcare systems.

## Conclusion

This multicenter retrospective study of Japanese patients with mHSPC who received Bic-CAB as an initial treatment elucidated the relevance of PSA dynamics in estimating long-term prognosis. The status of achievement of PSA nadir < 0.2 ng/mL and the range of PSA change before and after treatment may allow for the identification of patients with good 5- and 10-year survival rates. Further research is warranted to shape future recommendations that will enhance current treatment algorithms and improve patient outcomes.

## Data Availability

All data generated or analyzed during this study, which support the findings of this study, are included within this article and its supplementary information files. Researchers may access analyses not present in the manuscript from the corresponding author upon reasonable request. For the Astellas criteria on data sharing, see: https://clinicalstudydatarequest.com/Study-Sponsors/Study-Sponsors-Astellas.aspx.
